# Determinants of adherence and safety in fully autonomous home-based transcranial direct current stimulation for fibromyalgia

**DOI:** 10.3389/fpain.2026.1773425

**Published:** 2026-03-16

**Authors:** Wolnei Caumo, Lucas Bohrer Flores, Isabela Souza Fontoura, Graziele Borges Bueno, Paulo Robeto Stefani Sanches, Danton Pereira Silva, Iraci L. S. Torres, Felipe Fregni

**Affiliations:** 1 Post-Graduate Program in Medical Sciences, School of Medicine, Universidade Federal do Rio Grande do Sul (UFRGS), Porto Alegre, Brazil; 2 Laboratory of Pain and Neuromodulation, Hospital de Clínicas de Porto Alegre (HCPA), Porto Alegre, Brazil; 3 Pain and Palliative Care Service, Hospital de Clínicas de Porto Alegre (HCPA) and Department of Surgery, School of Medicine, Universidade Federal do Rio Grande do Sul (UFRGS), Porto Alegre, Brazil; 4 Laboratory of Biomedical Engineering, Hospital de Clínicas de Porto Alegre (HCPA), Porto Alegre, Brazil; 5 Laboratory of Pharmacology of Pain and Neuromodulation, Preclinical Investigations, Experimental Research Center, Hospital de Clínicas de Porto Alegre (HCPA), Porto Alegre, Brazil; 6 Laboratory of Neuromodulation and Center for Clinical Research Learning, Department of Physical Medicine and Rehabilitation, Spaulding Rehabilitation Hospital, Harvard Medical School, Boston, MA, United States

**Keywords:** adherence, feasibility, fibromyalgia, home-based-tDCS, safety, tDCS

## Abstract

**Background:**

Autonomous home-based transcranial direct current stimulation (HB-tDCS) is a scalable non-pharmacological intervention for chronic pain, but evidence under fully autonomous use remains limited.

**Objectives:**

To identify factors associated with adherence to fully autonomous HB-tDCS; to characterize the incidence, severity, and timing of adverse events under active and sham stimulation; and to examine if patient and clinical factors, including adverse events and clinical response, are associated with variability in adherence and safety outcomes.

**Design:**

Secondary pooled analysis of three double blind, sham-controlled randomized clinical trials.

**Subjects:**

A total of 234 women with fibromyalgia diagnosed according to the 2016 American College of Rheumatology criteria.

**Methods:**

Participants received a standardized autonomous HB-tDCS protocol (≥20 sessions). The primary outcomes were adherence and safety. We examined associations with adherence and adverse events using generalized linear mixed-effects models, adjusted for demographic, clinical, and psychosocial factors. We also explored whether the burden of adverse events explained the link between treatment allocation and adherence.

**Results:**

Overall adherence was 82.6% (95% CI, 81.6–83.6). Adherence rates were similar for the active (84.7%, 95% CI, 83.3–86.0) and sham groups (80.5%, 95% CI, 78.9–82.1). Older age (*β* = −0.024 sessions/year; 95% CI, −0.040 to −0.008) and greater central sensitization (*β* = −0.025 per point; 95% CI, −0.040 to −0.010) were associated with lower adherence. Higher depressive symptom severity was linked to higher adherence (*β* = 0.041 per point; 95% CI, 0.040–0.060). Most participants reported no adverse events (67.0%, 95% CI, 61.0–73.0), with no group differences (*χ*^2^ = 0.04, *p* = 0.83). Reported events were mostly mild and brief. Active stimulation led to more frequent tingling and erythema. Higher education (*β* = 0.078; 95% CI, 0.023–0.130) and depressive symptoms (*β* = 0.037; 95% CI, 0.020–0.050) were associated with more adverse event reports. Greater functional impairment was linked to fewer reported events (*β* = −0.003; 95% CI, −0.040 to −0.007).

**Conclusions:**

Autonomous home-based transcranial direct current stimulation was feasible, safe, and highly adhered to in fibromyalgia, with adherence and safety influenced by clinical and psychosocial factors, supporting scalable implementation in chronic pain.

## 1 Introduction

Fibromyalgia (FM) is characterized by widespread pain, fatigue, sleep disturbance, and cognitive difficulties (1). This condition leads to substantial healthcare use, reduced work productivity, and increased disability. For example, in a nationwide Danish cohort using health, labor, and social security registries, the estimated total societal cost was around €27,000 per patient annually, with financial losses beginning several years before diagnosis (2). Although international guidelines recommend a multimodal management strategy emphasizing non-pharmacological interventions, clinical practice remains predominantly pharmacological. Recent overviews and network meta-analyses show that approved pharmacological treatments achieve clinically meaningful pain reduction for only a minority of patients and demonstrate limited long-term benefit (3, 4).

Recent investigations have highlighted two main challenges: widespread use of many medications and poor medication adherence. Clifford-Faugère et al. (5) found that more than half of FM patients were taking 5 or more medications, whereas earlier studies reported an average of about 2 medications per patient (6–8). Large-scale observational studies also show high use of opioids and anti-inflammatory drugs among people with FM (9). However, treatment persistence is low; discontinuation rates range from 70% to 90% across drug classes (10–13). Together, these patterns reveal a gap between guidelines and real-world management, resulting in worse outcomes and higher healthcare costs (14, 15). As a result, non-pharmacological strategies are receiving increasing attention. Among these, transcranial direct current stimulation (tDCS) has emerged as a promising approach due to its ability to modulate cortical excitability and reorganize pain-related neural networks. Anodal stimulation is thought to induce subthreshold depolarization of the resting membrane potential, thereby increasing cortical excitability, whereas cathodal stimulation tends to promote hyperpolarization and reduce neuronal firing probability. These polarity-dependent effects can facilitate synaptic plasticity and modulate dysfunctional pain-related networks when stimulation is applied repeatedly (16). Current evidence supports Level A efficacy for depression and Level B for several neurological and pain conditions, including fibromyalgia (17–20). However, the need for repeated, supervised sessions is a major barrier. To address issues of access and continuity, home-based tDCS (HB-tDCS) systems have been developed (21–24).

The effectiveness and scalability of HB-tDCS depend on the availability of devices with robust monitoring and safety features—such as automatic session logging, dose verification, session limits, and lockout systems—as well as on standardized protocols and a clear understanding of patient-related factors that affect use (25). Supporting this, our clinical trials in fibromyalgia demonstrate the feasibility and safety of HB-tDCS (26–28). Despite this, key knowledge gaps persist. Specifically, it remains unclear which factors predict long-term adherence, what causes adverse effects in real-world conditions, and which patient profiles are most likely to benefit from the intervention. These unresolved questions hinder the development of personalized HB-tDCS strategies and broader implementation in fibromyalgia care. Most previous research on feasibility and safety has relied on remotely supervised or code-mediated protocols, which differ substantially from real-world autonomous use. Addressing this gap, the present study examines a fully autonomous, pre-programmed HB-tDCS model, in which patients independently initiate stimulation within a prescribed therapeutic window without research supervision. By integrating objective device logs with detailed clinical and psychosocial profiling, the study reconceptualizes adherence and adverse event reporting as behavioral phenomena shaped by patient characteristics rather than purely technical or device-related outcomes.

Guided by an implementation-oriented framework, this secondary pooled analysis of three randomized controlled trials aimed to: (i) identify clinical, behavioral, and psychosocial factors independently associated with adherence to fully autonomous home-based transcranial direct current stimulation; (ii) characterize the incidence, severity, and temporal patterns of adverse events under active and sham stimulation; and (iii) examine whether patient-related and clinical factors, including adverse event burden and clinical response, are associated with or account for variability in adherence and safety outcomes. Collectively, these objectives address a critical translational gap between supervised efficacy trials and real-world implementation.

## 2 Methods

### 2.1 Study design and data sources

We conducted a longitudinal analysis of three double-blind, sham-controlled randomized clinical trials registered at ClinicalTrials.gov, all employing a standardized tDCS protocol (27, 29, 30) (Table 1). The study protocols were approved by the Institutional Review Board at the Hospital de Clínicas de Porto Alegre (HCPA), Brazil (approval no. 2025-0346), and registered in the Brazilian national research ethics platform (CAAE: 93610825.2.0000.5327). Written informed consent was obtained from all participants.

**Table 1:** Descriptive characteristics of the randomized double-blind controlled clinical trial using the home-based-tDCS protocol (*n* = 234).

Clinical Trial Identifier	Diagnosis	Time period	Study Design	Number Of Sessions	tDCS Current Intensity (mA)	Electrode Montage		tDCS Duration (min)	Paired activity
**(Study 1).**	Fibromyalgia (*n* = 102)	September 2019 to November 2022	Sham-controlled RCT	20	2	a-tDCS –l-DLPFC a-M1	cathode r-DLPFC cathode M1-SO	20	-
NCT0									
3843203									
Caumo, 2024									
**(Study 2).**	Fibromyalgia (*n* = 112)	May 2023 to December 2024	Sham-controlled RCT	20	2	a-tDCS –l-DLPFC	cathode r-DLPFC	20	Exercice and neuroeducation in pain
NCT									
05845528									
Caumo, 2020									
**(Study 3)**	Fibromyalgia (*n* = 20)	January 2017 to July 2018	Sham-controlled RCT	60	2	a-tDCS l–DLPFC	cathode r-DLPFC	20	——
NCT									
02652988									
Britzcke 2019									

M1, primary motor cortex; SO, supraorbital region; RCT, randomized clinical trial.

The pooled dataset comprised three randomized controlled trials that differed in stimulation target, number of sessions, and treatment duration. One trial applied stimulation over the primary motor cortex (M1) and the left dorsolateral prefrontal cortex (DLPFC), whereas two trials used a bifrontal montage targeting the left DLPFC. Protocols also varied in the total number of sessions (20 vs. 60) and in overall intervention duration. These differences reflect distinct therapeutic rationales: M1 stimulation primarily targets nociceptive modulation, while DLPFC stimulation may influence the affective and cognitive dimensions of pain.

### 2.2 Participants

We included literate women aged 18–75 years, right-handed, recruited from the Chronic Pain Outpatient Clinic of HCPA, Brazil, and via media advertisements. Fibromyalgia was diagnosed according to the 2016 revisions of the American College of Rheumatology (ACR) criteria, requiring: (i) a Widespread Pain Index (WPI) ≥ 7 with a Symptom Severity Score (SSS) ≥ 5, or WPI 4–6 with SSS ≥ 9; (ii) generalized pain in ≥ 4 of 5 regions; (iii) symptoms persisting ≥3 months; and (iv) diagnosis irrespective of comorbidities. Eligible patients reported daily disability due to FM for ≥3 months, pain intensity ≥50 mm on a 0–100 Numerical Pain Scale (NPS), and agreed to maintain stable antidepressant or anticonvulsant doses. Exclusion criteria included contraindications to noninvasive brain stimulation, autoimmune, neurological or oncological disorders, uncompensated clinical conditions (e.g., ischemic heart, renal, or liver disease), and cannabis or other recreational psychotropic drug use within the prior 6 months.

### 2.3 Dependent variables and assessments

#### 2.3.1 Outcomes

The primary outcomes were adherence and safety under fully autonomous home-based tDCS.

Adherence was defined as engagement with the prescribed stimulation protocol and was objectively quantified using device-generated logs. Safety was assessed by the incidence and severity of adverse events (AEs). Adherence was operationalized according to treatment conditions. In the active stimulation (a-tDCS) group, adherence was quantified as the number of valid sessions, defined as sessions with effective current delivery lasting ≥10 min, the minimum duration required to induce sustained neuroplastic effects (16, 31). In the sham stimulation (s-tDCS) group, adherence was quantified as the number of sessions initiated and registered by the device, reflecting engagement with the protocol in the absence of sustained current delivery. Importantly, adherence and session validity represent distinct constructs in this study. Adherence reflects behavioral engagement with the prescribed protocol and was operationalized as the number of sessions initiated, whereas session validity reflects the technical adequacy of stimulation delivery, defined by impedance values within predefined safety thresholds and minimum stimulation duration. Because sustained current delivery does not occur during sham stimulation by design, session validity cannot be equivalently defined across groups. Accordingly, adherence was intentionally operationalized as the number of sessions initiated in both active and sham conditions to ensure behavioral comparability, while impedance-based validity metrics were analyzed descriptively and restricted to the active arm as indicators of technical quality.

#### 2.3.2 Report on adverse events

Participants recorded adverse effects in a diary after each home-based tDCS session, completed a final questionnaire on the occurrence, and self-rated severity of side effects. Potential effects included headache, scalp pain, tingling, itching, burning, erythema, somnolence, difficulty concentrating, and acute mood changes, rated as mild, moderate, or severe (32). Adverse events (AEs) were defined according to the Common Terminology Criteria for Adverse Events (CTCAE) as any undesirable outcome following a medical intervention, regardless of causal relationship, and were distinguished from side effects (33, 34). AE severity was classified into five grades: grade 1 (mild, self-limited symptoms such as erythema or tingling), grade 2 (moderate, requiring local treatment), grade 3 (severe, clinically significant or requiring hospitalization), grade 4 (life-threatening), and grade 5 (death) (23).

#### 2.3.3 Assumptions regarding dose–response relationship

The dose of transcranial electrical stimulation (TES) is defined by stimulation parameters that determine the induced electric field (V/m) (35). For tDCS, dosage is commonly expressed as charge density, calculated as current × duration/electrode area. A 2 mA stimulation with standard 35 cm^2^ electrodes yields charge densities of ∼170–480 C/m^2^ (36). In addition to charge density, electrode–skin impedance is a relevant outcome measure, as it reflects electrode contact quality, influences current distribution, and provides an indirect indicator of safety and stimulation reliability (37, 38).

### 2.4 Independent variables

#### 2.4.1 Exposure variables include the intervention with a-tDCS or s-tDCS

Participants were exposed to a standardized home-based tDCS (HB-tDCS) (details about the protocol in Supplemental material) program using a device developed by the Biomedical Engineering Department of HCPA and the Laboratory of Pain and Neuromodulation (ANVISA N° 80079190028), Porto Alegre, Brazil. Each participant received a kit including the device, neoprene cap, electrodes, and saline solution. Electrodes (35 cm^2^, saline-soaked sponges) were fixed to the cap to ensure stability, with placement according to the EEG 10–20 system (anode F3/cathode F4 or anode C3/cathode Fp2). Stimulation consisted of 2 mA for 20 min with 20-s ramp-up/down, five sessions per week. Sham stimulation delivered only brief currents (30 s at baseline, 10, and 20 min) to mimic sensations without sustained current. Devices were preprogrammed to enforce ≥16 h between sessions and continuously monitored impedance, automatically interrupting if thresholds were exceeded. Further details regarding the HB-tDCS protocol are provided in the Supplementary Material S2.

The first session was supervised in the center to train cap fitting and device handling; subsequent sessions were home-based with optional remote support. Weekly contacts reinforced adherence. Device logs (time, duration, intensity, impedance) and patient diaries were used for compliance and adverse event monitoring. A minimum of 10 min within acceptable impedance range (≈8–4 kΩ at onset, 3–2 kΩ during stimulation) was required to consider a session valid, consistent with evidence that neuroplastic after-effects occur after ≥10 min of stimulation (16, 31, 37).

### 2.5 Clinical and psychological measurements

*B-PCP-S—Brazilian Profile of Chronic Pain:* Screen assesses multidimensional pain index across severity (0–32), disability (0–36), and emotional burden (0–25), with a total score of 0–93. Higher scores indicate greater disability or distress. Changes were analyzed at baseline, post-treatment, and three-month follow-up (39).
*FIQ—Fibromyalgia Impact Questionnaire* evaluates the impact of fibromyalgia on quality of life across function (9 items), overall impact (2 items), and symptoms (10 items). Items are scored according to standardized procedures, yielding a total score from 0 to 100 (40).
*Psychological measures and central sensitiz*ati*on symptoms* were evaluated using the Central Sensitization Inventory, Brazilian version (41). Depressive symptoms were measured with the Beck Depression Inventory-II (42). Pain-related catastrophic thinking was assessed with the Pain Catastrophizing Scale, Brazilian version (43).
Demographics, comorbidities, and analgesic use—Collected using a standardized questionnaire. Concomitant medications were permitted, and intake was recorded in diaries.

### 2.6 Efforts to address potential sources of bias

This study pooled original data from three randomized, double-blind, sham-controlled clinical trials, minimizing selection and performance bias. Pre-programmed devices prepared solely by biomedical engineers, preventing access to treatment assignment, ensured allocation concealment. Objective measures—automatic session logs, impedance monitoring, and lockout systems—reduced reporting and adherence bias. Adverse events were captured using standardized forms based on the Common Terminology Criteria for Adverse Events (CTCAE), limiting variability in classification. Finally, multivariable mixed-effects models adjusted for relevant clinical and psychosocial covariates to control for confounding.

### 2.7 Statistical analysis

Continuous variables were compared using independent-sample t tests, and categorical variables using Fisher's exact or chi-square tests. Normality was assessed with the Shapiro–Wilk test. Primary outcomes (number and severity of adverse events and number of tDCS sessions) were analyzed using linear mixed-effects models for repeated measures (LMM), with treatment group, time, and the group-by-time interaction specified as fixed effects and a random intercept for participants to account for within-subject correlation. Because data were pooled from three randomized trials with different stimulation protocols, trial and stimulation target (montage) were included as fixed-effect covariates to control for protocol-level differences. To formally evaluate whether treatment effects varied across studies or stimulation targets, Treatment × Trial and Treatment × Montage interaction terms were tested, allowing assessment of potential heterogeneity attributable to protocol characteristics rather than individual-level variability. All models including trial and montage effects and their interaction terms are reported in the Supplementary Material Tables S1–S4.

Predictors of adherence (number of valid sessions) were examined using linear mixed-effects models including educational level, depressive symptoms, central sensitization, age, and multidimensional pain index domains (pain intensity, disability, and emotional burden) as covariates. Multicollinearity was assessed prior to model fitting, and pain catastrophizing was not included due to collinearity with multidimensional pain index domains, as indicated by variance inflation factor and tolerance criteria.

As a sensitivity analysis, adherence was alternatively defined as the number of sessions initiated (device-recorded attempts), independent of impedance or stimulation duration, to provide a fully comparable behavioral metric across active and sham conditions. Associations were compared with the primary adherence models based on valid sessions in the active group. Additional sensitivity analyses were conducted including trial as a fixed effect and using stratified models by stimulation target (M1 vs. DLPFC) and protocol duration (20-session vs. 60-session studies) to assess the consistency of associations across protocols Supplementary Material Tables S5–S8.

Exploratory mediation analyses were conducted to examine whether cumulative adverse event severity accounted for the association between treatment allocation and adherence, defined as the number of valid stimulation sessions. Treatment allocation was specified as the independent variable, cumulative adverse event severity as the mediator, and adherence as the outcome. Direct, indirect, and total effects were estimated using regression-based models with bootstrap resampling to derive 95% confidence intervals. All models were adjusted for baseline depressive symptoms, pain catastrophizing, and fibromyalgia severity by FIQ.

A two-sided significance level of 0.05 was adopted. When appropriate, Bonferroni corrections were applied, and paired t tests were conducted within groups. All analyses were two-tailed with a 5% significance level and performed using SPSS version 22.0. Mediation analyses were conducted in R using the mediation package, with indirect effects estimated via nonparametric bootstrapping and bias-corrected 95% confidence intervals.

## 3 Results

### 3.1 Study population and baseline characteristics

A total of 414 patients were screened for eligibility. Of these, 180 were excluded due to chronic diseases or contraindications to tDCS (*n* = 131), COVID-19 infection or persistent sequelae (*n* = 28), lockdown-related restrictions (*n* = 10), or failure to meet diagnostic criteria (*n* = 21). The remaining 234 patients were randomized to either active tDCS (a-tDCS; *n* = 134) or sham tDCS (s-tDCS; *n* = 100). Stimulation was delivered over the left dorsolateral prefrontal cortex (l-DLPFC) or the primary motor cortex (M1), depending on the trial protocol. In the bifrontal DLPFC trial, 112 women were randomized, with 10 withdrawals occurring after at least 10 stimulation sessions. In the DLPFC–M1 trial, 102 participants were randomized: 32 to a-tDCS over the DLPFC, 32 to a-tDCS over the M1, 17 to s-tDCS over the DLPFC, and 17 to s-tDCS over the M1; five withdrawals were observed (three in the a-tDCS group and two in the s-tDCS group). In the 60-session study, 20 participants were equally allocated to a-tDCS or s-tDCS.

The final analytical sample comprised 234 participants who received at least one stimulation session: 176 underwent DLPFC stimulation (114 a-tDCS and 62 s-tDCS) and 58 underwent M1 stimulation (32 a-tDCS and 26 s-tDCS). Baseline demographic, clinical, and psychosocial characteristics of the study population are presented in Table 2.

**Table 2:** Epidemiological and clinical characteristics at baseline, according to the treatment group, values are given as the mean (SD) or frequency (*n* = 234).

Characteristics	Total sample (*n* = 234)	sham-tDCS (*n* = 100)	a-tDCS (*n* = 134)	*p*-value
Age (years)	48.82 (9.49)	48.96 (10.01)	49.15 (8.91)	0.76
Formal education (years)	12.53 (3.73)	13.01 (3.70)	12.27 (3.89)	0.08
Working condition				0.37
Working	133 (56.83%)	55 (41.35%)	78 (58.64%)	
Unemployed	49	23	26	
Health license	34	17	17	
Retired	12	5	7	
Smoking (yes/no)	44/190	23/77	21/113	0.12
Alcohol use (yes/no)	31/203	16/84	15/119	0.18
Diagnosis of psychiatric disorder (yes/no)				
Panic Disorder (yes/no)	8/226	3/97	5/129	1
Major Depression Disorder (yes/no)	72/162	28/72	44/89	0.49
Anxiety Disorder (yes/no)	55/179	19/81	36/98	0.21
Analgesic medication use				
Opioid analgesic (yes/no)	38/196	15/85	23/111	0.43
Analgesic non-opioid (yes/no)	134/100	56/44	82/52	0.26
Use drug active on the nervous system (yes/no)*				
Selective Serotonin Reuptake Inhibitor (yes/no)	46/188	15/85	31/103	0.09
Duloxetine(yes/no)	81/153	29/71	52/82	0.08
Tricyclic antidepressant (yes/no)	30/204	12/88	18/115	0.42
Pregabaline/Gabapentine (yes/no)	46/188	24/76	42/92	0.15
History of chronic disease (yes/no)	147/87	60/40	87/47	0.53
Hypertension (yes/no)	89/145	39/61	50/84	
Diabetes Mellitus (yes/no)	31/203	13/87	18/116	
Thyroid disease (yes/no)	11/223	4/96	7/127	
Asthma (yes/no)	39/197	16/86	23/111	
American College of Rheumatology (ACR-2016)- fibromyalgia criteria	21.38 (3.87)	21.24 (3.91)	21.64 (3.89)	0.46
SSS—Symptom Severity Scale	9.31 (1.77)	9.34 (1.69)	9.32 (1.75)	0.91
WPI—Widespread Pain Index	12.18 (2.99)	12.00 (2.91)	12.46 (3.09)	0.27
Central Sensitization inventory	65.58 (15.06)	63.93 (14.35)	65.67 (15.15)	0.38
Beck Depression Inventory—BDI-II	26.88 (11.94)	25.95 (11.39)	25.99 (11.82)	0.98
Pain Catastrophizing Scale—PCS	35.19 (11.20)	37.17 (10.71)	35.97 (10.71)	0.58
Numerical Pain Scale (0–10)-	6.69 (2.05)	6.54 (2.21)	7.31 (1.80)	0.004
Fibromyalgia Impact Questionnaire (FIQ)—pre intervention	72.62 (13.91)	71.10 (15.48)	72.70 (13.03)	0.40
Fibromyalgia Impact Questionnaire (FIQ) post intervention	58.60 (16.95)	62.82 (16.74)	55.57 (16.51)	0.003
Multidimensional B-PCP-S—Pre-intervention	73.15 (13.13)	72.61 (12.69)	72,83 (13.36)	0.90
Multidimensional B-PCP-S—Post-intervention	61.46 (16.16)	64.87 (15.39)	59.07 (16.39)	0.01

* Patients could have none or more than one diagnostic according.

### 3.2 Feasibility and global adherence to autonomous HB-tDCS

**Figure 1:**
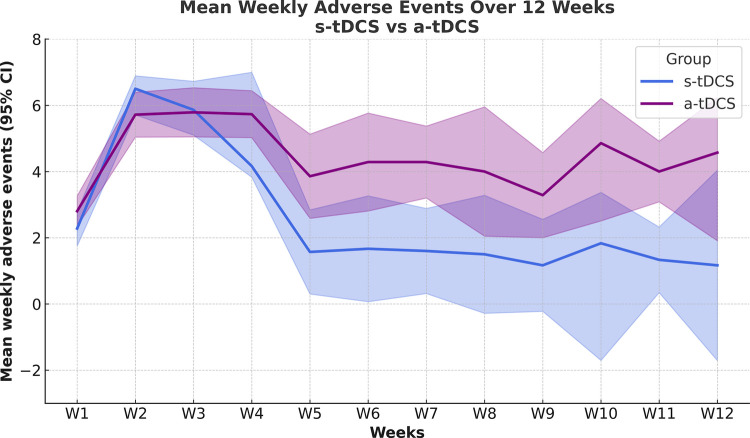
Weekly adherence to home-based tDCS by treatment group over the intervention period. The *x*-axis represents study week, and the *y*-axis represents the mean number of device-recorded stimulation sessions per week. Active (a-tDCS, blue) and sham (s-tDCS, lilac) groups are shown separately. Values correspond to participants with available device logs at each time point Shaded areas indicate the 95% confidence intervals (CI) for each group. From week 5 onward, the protocol included only the subsample of 20 participants randomized 1:1 (10 in the s-tDCS group and 10 in the a-tDCS group), which is reflected in the widening of confidence intervals at later time points. Values correspond to participants with available device logs each week. Blue and lilac curves represent the s-tDCS and a-tDCS groups, respectively.

The longitudinal trajectory of adherence across treatment weeks is illustrated in Figure 1. Across the three randomized clinical trials, a total of 5,480 stimulation sessions were scheduled. Of these, 4,541 sessions were completed, resulting in an overall adherence rate of 82.6%. Adherence was marginally higher in the active stimulation group compared with the sham group. Adherence was defined as the number of sessions performed, irrespective of impedance, whereas treatment validity required an impedance ≤10 k*Ω* to ensure effective current delivery. In the active tDCS (a-tDCS) group, 3,080 sessions were planned, and participants completed 2,609 sessions, corresponding to an adherence rate of 84.7%. In the sham tDCS (s-tDCS) group, 2,400 sessions were planned and 1,932 were completed, yielding an adherence rate of 80.5%. Together, these values correspond to the overall adherence rate of 82.6%. Objective device-generated impedance logs demonstrated a high proportion of valid sessions across both treatment arms. In the a-tDCS group, 156 sessions (6.0%) exceeded the predefined impedance threshold of 10 kΩ and were excluded, resulting in 2,453 valid stimulation sessions.

Adherence was defined as the number of sessions initiated and recorded by the device, irrespective of impedance, whereas treatment validity required impedance ≤10 kΩ and minimum stimulation duration. Thus, while impedance-defined validity was assessed only in the active stimulation arm as a technical quality indicator, adherence was defined uniformly across groups as behavioral engagement with the protocol, ensuring comparability between active and sham conditions.

**Figure 2:**
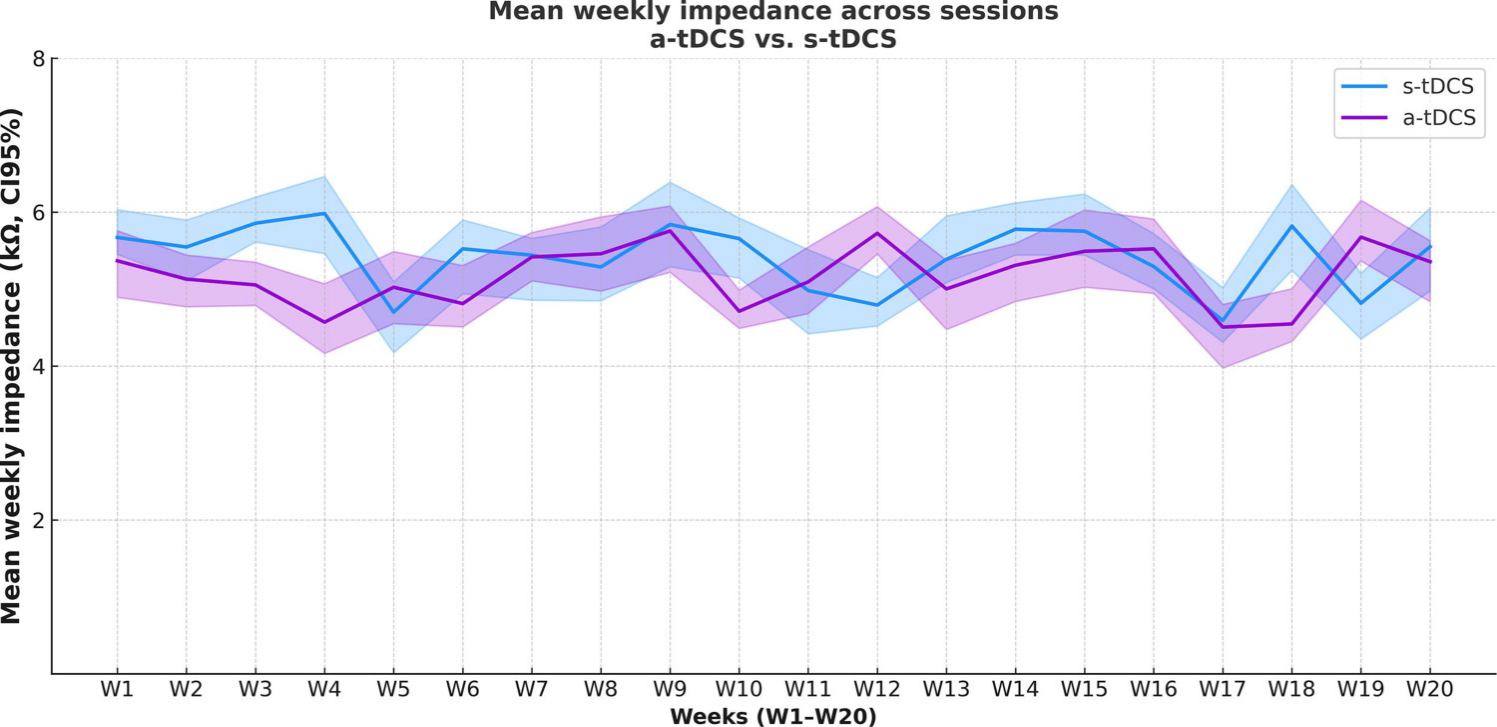
Mean electrode–skin impedance values with 95% confidence intervals (CI) across weekly sessions (S1–S20) in the sham and a-tDCS groups. In the Sham group, impedance was measured at three points within each session (at the beginning, after 10 min, and after 20 min. In the a-tDCS group, the mean was calculated across the entire session, providing a single measure of impedance stability throughout stimulation. The vertical axis was limited to 0–8 kΩ for better visualization.

Session technical quality over time, assessed by mean contact impedance, is presented in Figure 2. Mean impedance was 5.17 (SD 6.07) *Ω* in the a-tDCS group and 5.38 (SD 1.74) Ω in the s-tDCS group, with 95% confidence intervals displayed across sessions (S1–S12) for both groups. In the sham condition, impedance was recorded at baseline, 10 min, and at the end of stimulation, whereas in the a-tDCS group impedance values were averaged across the entire session. Generalized linear mixed-effects model (GLMM) analysis showed no significant main effects of group (*F* = 6.48, *p* = 0.36) or time (*F* = 0.02, *p* = 0.88), and no significant group ×  time interaction (*F* = 0.32, *p* = 0.81).

### 3.3 Determinants of adherence: clinical and psychosocial predictors

Table 3 presents the multivariable mixed-effects models examining clinical and psychosocial predictors of adherence. Older age and greater central sensitization were independently associated with lower adherence, whereas higher baseline depressive symptom severity was associated with greater adherence. In contrast, a significant interaction between the multidimensional pain index and treatment allocation indicated greater protocol persistence among participants with higher symptom burden in the sham group.

**Table 3:** GLMM examining factors associated with treatment adherence (number of sessions completed (*n* = 234).

Outcome: number of weekly sessions of tDCS across the treatment						
	Beta	SEM	gl	*t*	*P*	CI 95%
*Intercept*	4.590836	.691260	607.732	6.641	.000	(3.23 to 5.94)
Sham-tDCS/ Active-tDCS0^Reference^	−.808729	.316723	176.516	−2.553	.012	(−1.43 to −0.18)
Formal education (ys)	.013752	.021203	623.602	.649	.517	(−.028 to 0.06)
Age (ys)	−.023987	.008099	622.390	−2.962	.003	(−.04 to −0.008)
Beck Depressive Inventory -II	.041268	.009013	620.717	4.579	.000	(.04 to 0.06)
Central Sensitization inventory	−.025301	.007294	621.886	−3.469	.001	(−.04 to −0.01)
Multidimensional pain index	−.001286	.006172	624.008	−.208	.835	(−.01 to 0.01)
Sham-tDCS vs. Multidimensional pain index	.027779	.011882	620.532	2.338	.020	(.004 to 0.05)
Active-tDCS vs. Multidimensional pain index	0^Reference^					

The multidimensional pain index comprised three domains—pain severity, disability, and emotional burden—deriv*e*d from the Brazilian Profile of Chronic Pain: Screen (B-PCP-S). Pain catastrophizing was not included in this model due to collinearity with B-PCP-S.

Sensitivity analyses defining adherence exclusively as the number of sessions initiated in both groups yielded consistent effect estimates and did not materially alter the associations observed in the primary models (Supplementary Tables S1–S4). Additional sensitivity analyses including trial and stimulation target as fixed effects showed that the association between treatment allocation and adherence remained significant, whereas no independent effects of trial or stimulation target were observed. No significant treatment-by-trial or treatment-by-target interactions were detected, indicating absence of protocol-related heterogeneity.

Further models adjusting for baseline depressive symptoms, change in depressive symptoms, or both yielded consistent treatment effects, supporting the robustness of the main findings. Stratified analyses by trial and an exploratory comparison between DLPFC and M1 stimulation within the factorial study also showed concordant results. Detailed results are provided in Supplementary Tables S1–S4.

### 3.4 Safety profile and incidence of adverse events

Approximately two-thirds of participants did not report any adverse events during the intervention period. The overall distribution was similar between groups: 67.0% vs. 66.0% reported no events, 17.8% vs. 19.9% reported mild symptoms, and 15.2% vs. 14.2% reported moderate-to-severe events, respectively. When dichotomized, 33.0% (s-tDCS) and 34.0% (a-tDCS) reported at least one adverse event (AE), with no significant difference (*χ*^2^ = 0.04, df = 1, *p* = 0.83). Most events were mild and transient, supporting a comparable safety profile between groups. Significant group effects emerged only for tingling, redness, and concentration difficulty: tingling and redness were more frequently mild in the a-tDCS group, whereas their absence predominated in the s-tDCS group; conversely, concentration difficulty was more often absent in the a-tDCS group, while severe cases were slightly higher in the s-tDCS group. No between-group differences were found for headache, itching, burning, sleepiness, or mood changes. The frequency and distribution of adverse events by treatment arm are presented in Table 4.

**Table 4:** Adverse events presented as percentage, and the incidence or severity of side events classified as absence, mild, moderate, and severe (*n* = 234).

Symptoms	Treatments	Absence	Mild	Moderate	Severe	*P*-value
Headache						
	s-tDCS (*n* = 100)	61.2%	16.7%	18.8%	3.3%	0.65
	*a*-tDCS (*n* = 134)	59.1%	19.8%	17.6%	3.6%	
Tingling						
	*s*-tDCS (*n* = 100)	68.5%	20.0%	8.5%	3.1%	0.002
	a-tDCS (*n* = 134)	58.6%*	29.1%*	10.5%	1.8%	
Itching						
	s-tDCS (*n* = 100)	56.9%	29.4%	11.5%	2.1%	0.38
	a-tDCS (*n* = 134)	53.6%	29.2%	15.3%	2.0%	
Burning						
	s-tDCS (*n* = 100)	63.5%	24.5%	10.4%	1.6%	0.69
	a-tDCS (*n* = 134)	61.2%	26.0%	11.7%	1.1%	
Caumo, 2020						
	s-tDCS (*n* = 100)	91.8%*	4.5%	2.8%	0.9%	0.02
	a-tDCS (*n* = 134)	87.7%	9.3%*	2.7%	0.4%	
Caumo, 2020						
	s-tDCS (*n* = 100)	55.1%	20.0%	18.8%	6.1%	0.21
	a-tDCS (*n* = 134)	58.5%	22.1%	14.9%	4.4%	
Concentration difficulty						
	s-tDCS (*n* = 100)	65.2%	16.7%	12.9%	5.2%	0.02
	a-tDCS (*n* = 134)	70.3%*	12.8%	14.6%	2.3%	
Mood changes						
	s-tDCS (*n* = 100)	74.1%	10.4%	10.4%	5.2%	0.15
	a-tDCS (*n* = 134)	78.6%	10.7%	7.3%	3.4%	

The bold asterisk indicates a statistically significant difference between the active and sham groups (*p* < 0.05).

### 3.5 Determinants of adverse event reporting

**Figure 3:**
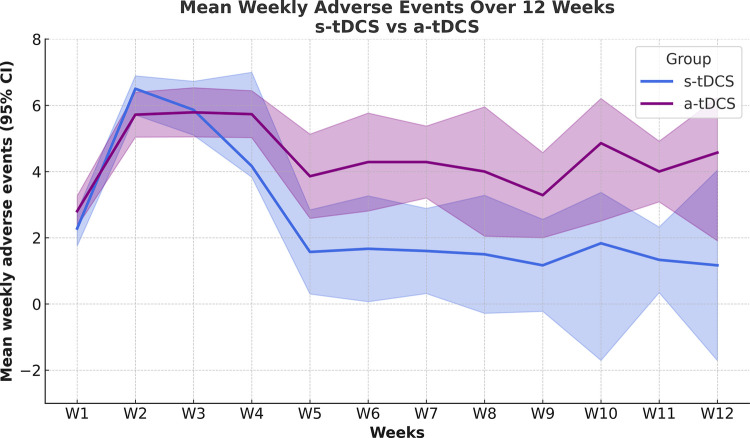
Mean number of weekly adverse events by treatment group over the intervention period. The *x*-axis represents study week, and the *y*-axis represents the mean number of reported adverse events per week. Active (a-tDCS, blue) and sham (s-tDCS, lilac) groups are shown. Shaded areas indicate the 95% confidence intervals (CI). From week 5 onward, data reflect the subsample of participants enrolled in the 60-session protocol.

Temporal trends in adverse events reporting across treatment weeks are shown in Figure 3. The weekly mean count of adverse events reported in the s-tDCS and a-tDCS groups over the full treatment period. From week 5 onward, the results reflect a subsample of 20 patients who were randomly allocated to each group. The GMM analysis showed that active tDCS (a-tDCS) significantly increased the number of AEs over the 12-week treatment period (*F* = 45.0, *p* < .0001). Significant effects of time (*F* = 19.32, *p* = .001) and time ×  treatment interaction (*F* = 4.90, *p* = .001) were also observed.

Table 5 presents the analysis of predictors of adverse event reporting, showing that psychosocial factors were more strongly associated with reported events than technical stimulation parameters. Higher educational attainment and greater baseline depressive symptoms were associated with increased adverse event reporting, whereas greater functional impairment was associated with fewer reported events.

**Table 5:** GLMM examining factors associated with number of adverse events across treatment (*n* = 234).

Outcome: Number of adverse events across treatment						
	Beta	SEM	gl	*t*	*P*	CI 95%
Intercept	4.58	1.317	20.84	3.48	0.002	(1.84 to 7.23)
S-tDCS/ a-tDCS	−3.61	1.649	11.07	−2.19	0.051	(−7.24 to 0.02)
Formal education (ys)	0.078	0.027	298.26	2.81	0.005	(.023 to 0.13)
Age (ys)						
Beck Depressive Inventory -II	0.037	0.009	175.89	3.98	0.000	(0.02 to 0.05)
Central Sensitization inventory	0.007	0.009	161.56	0.80	0.421	(−0.01 to 0.03)
Multidimensional pain index	−0.003	0.009	312.68	−2.76	0.006	(−0.04 to −0.007)

The multidimensio*n*al pain index was entered as a global score derived from the Brazilian Profile of Chronic Pain: Screen (B-PCP-S). Pain catastrophizing was not included in this model due to collinearity with B-PCP-S.

### 3.6 Linking adverse event severity and adherence: implementation insights

**Figure 4:**
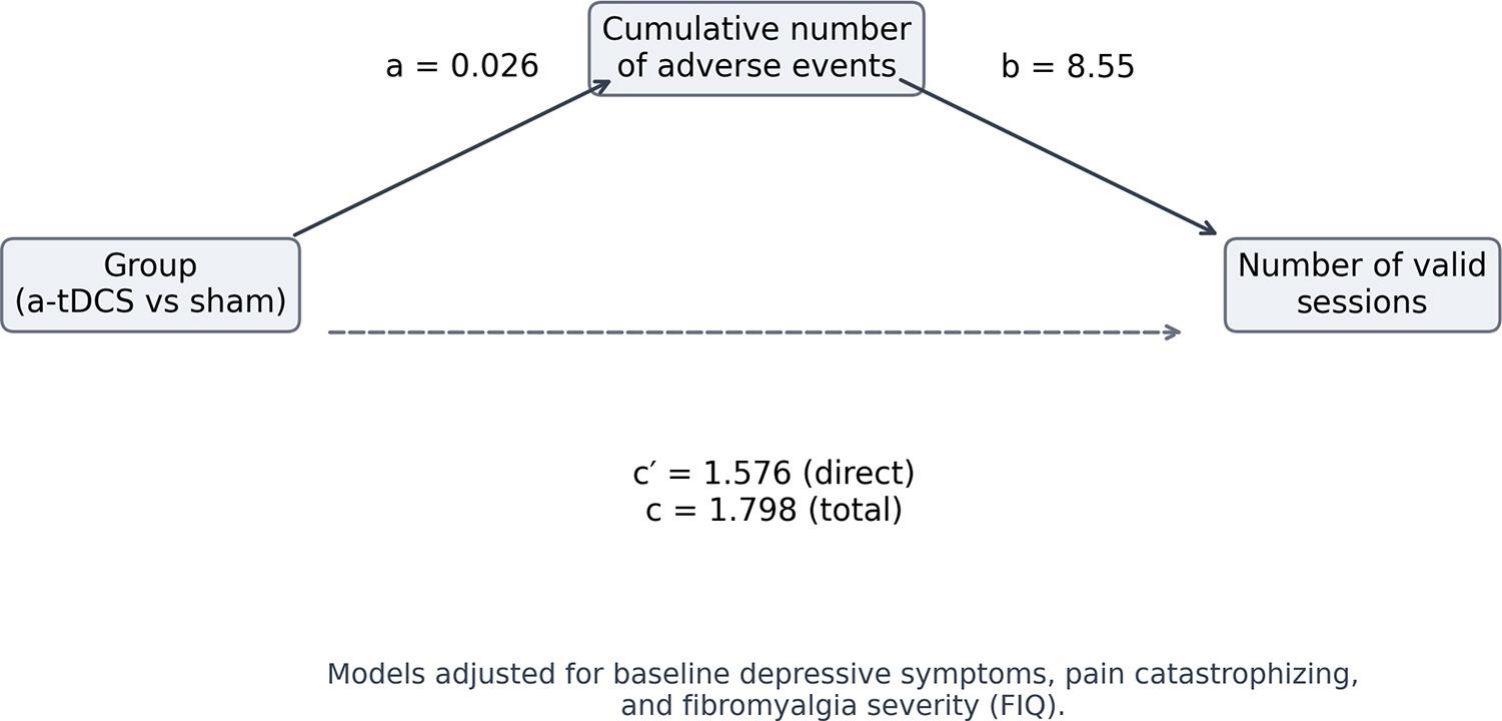
Mediation model linking treatment allocation, cumulative adverse event severity**,**and adherence. Exploratory mediation analysis examining whether the cumulative number of adverse events, mediated the association between treatment allocation (active tDCS vs sham) and adherence, defined as the total number of valid stimulation sessions. ***Path* a** represents the association between treatment group and adverse event burden; ***path* b** represents the association between adverse event burden and adherence, adjusted for treatment group; ***path* c**′ represents the direct effect of treatment group on adherence after accounting for adverse event burden, and ***path* c** represents the total effect. All models were adjusted for baseline depressive symptoms, pain catastrophizing, and fibromyalgia severity (FIQ). Regression coefficients are shown next to each path.

Exploratory analyses indicated that greater cumulative adverse event severity was associated with higher protocol engagement, whereas treatment allocation alone did not predict adherence (Figure 4). Treatment allocation was not associated with cumulative adverse event severity (***path a:*** *β* = 0.026, 95% CI −0.009 to 0.060, *p* = 0.16). In contrast, greater adverse event severity was independently associated with a higher number of valid sessions after adjustment for baseline depressive symptoms, pain catastrophizing, and fibromyalgia severity (***path b:*** *β* = 8.55, 95% CI 2.16 to 15.43, *p* = 0.032). The direct effect of treatment allocation on adherence was not significant after accounting for adverse event severity (***path c***′***:*** *β* = 1.58, 95% CI −0.47 to 3.57, *p* = 0.13), and the total effect showed only a non-significant trend (***path c***: *β* = 1.80, 95% CI −0.21 to 3.78, *p* = 0.084). The bootstrap-estimated indirect effect was small and not significant (***a*** ***×*** ***b***: *β* = 0.22, 95% CI −0.08 to 0.61), indicating that cumulative adverse event severity did not mediate the relationship between treatment allocation and adherence. In adjusted models, baseline depressive symptoms were positively associated with the number of valid sessions (*β*std = 0.18, 95% CI 0.04 to 0.31), whereas greater fibromyalgia severity was inversely associated with adherence (*β*std = −0.16, 95% CI −0.29 to −0.03). Pain catastrophizing was not independently associated with adherence (*β*std = −0.05, 95% CI −0.18 to 0.08).

## 4 Discussion

In this pooled analysis of 234 women with fibromyalgia drawn from three randomized controlled trials, we demonstrated that fully autonomous home-based transcranial direct current stimulation (HB-tDCS) is feasible, safe, and associated with high levels of adherence. A total of 4,541 of 5,480 programmed sessions were completed, corresponding to an overall adherence rate of 82.6%. A central novelty of this study lies in the evaluation of a fully autonomous, pre-programmed HB-tDCS system equipped with automatic session logging, impedance monitoring, and dose control, allowing objective verification of both adherence and safety without continuous researcher mediation. Beyond feasibility, these findings advance the field by reframing adherence and safety in home-based neuromodulation as behavioral and psychosocial phenomena rather than purely technical outcomes. This implementation-oriented perspective aligns with established frameworks emphasizing that patient engagement, contextual factors, and individual symptom perception critically shape real-world effectiveness beyond device performance alone (44, 45). By integrating objective device-generated data with detailed clinical and psychosocial profiling, the present study provides a more ecologically valid understanding of HB-tDCS implementation in routine care.

This study further contributes novel insights by identifying specific clinical and psychosocial factors associated with adherence, a dimension that has been insufficiently explored in prior HB-tDCS research (46, 47). Older age and greater central sensitization were independently associated with lower adherence, whereas higher baseline depressive symptom severity was associated with greater adherence. These findings suggest that patients who are older or exhibit higher levels of central sensitization may benefit from tailored support strategies when autonomous neuromodulation is prescribed. Although the regression coefficients appear numerically small when expressed per unit of the predictor, these estimates represent incremental changes in weekly adherence that may accumulate across multi-week protocols, resulting in meaningful differences in cumulative stimulation dose and overall treatment exposure. In contrast, higher baseline depressive symptom severity was associated with greater engagement with the HB-tDCS protocol, and individuals with greater multidimensional pain index demonstrated higher protocol persistence under sham stimulation, indicating that perceived symptom burden may reinforce engagement independently of physiological stimulation effects. These findings highlight the relevance of patient motivation, perceived need for care, and symptom burden in shaping adherence behavior. Although depressive symptoms are commonly associated with poorer treatment adherence in pharmacological studies (48), in this context greater depressive symptom severity was associated with higher engagement with the HB-tDCS protocol. Importantly, participants were primarily seeking relief from chronic pain, and although depression is a highly prevalent comorbidity in fibromyalgia, pain-related suffering tends to be the main driver of treatment-seeking behavior. Thus, greater psychological distress may reflect a higher perceived need for care and stronger motivation to persist with a potentially beneficial intervention. In neuromodulation and placebo research, expectations and sensory experiences are known to reinforce perceived treatment credibility and sustained engagement with interventions (49, 50). Moreover, as tDCS remains a relatively novel and technology-based intervention for many patients, a potentially beneficial new therapeutic approach such as tDCS may increase expectations of improvement and, consequently, promote greater engagement and adherence to treatment. Similar patterns have been described in behavioral and device-based interventions, in which symptom burden and perceived need for care may increase—rather than reduce—protocol adherence (44, 45, 51).

The observed safety profile further supports the translational relevance of fully autonomous HB-tDCS. Approximately two-thirds of participants reported no adverse events, and most reported events were mild and transient, with no differences in overall incidence between active and sham stimulation. Importantly, adverse event reporting did not result in treatment discontinuation, reinforcing the distinction between event occurrence and clinically meaningful intolerance, as emphasized in established safety frameworks for noninvasive brain stimulation (32, 37). Moreover, exploratory mediation analyses indicated that cumulative adverse event severity did not mediate the association between treatment allocation and adherence. While the main mixed-effects models demonstrated a significant pooled treatment effect on adherence, additional analyses were conducted to explore potential heterogeneity across stimulation targets and protocols. Montage (DLPFC vs. M1) was not associated with adherence, and Treatment × Montage interaction terms were not significant in pooled or within-trial models, indicating that stimulation target did not modify the treatment effect. Trial-stratified analyses showed the same direction of effect across studies, although some within-trial estimates did not reach statistical significance, likely reflecting reduced power rather than true absence of effect. Overall, these findings support the robustness of the pooled results despite protocol differences across trials. Importantly, these analyses address feasibility and implementation consistency rather than physiological efficacy of specific montages, and therefore should not be interpreted as evidence of neurobiological equivalence between stimulation targets. Instead, they indicate that behavioral engagement with fully autonomous HB-tDCS protocols is stable across different technical configurations, supporting scalability from an implementation perspective.

Consistently, the active stimulation group reported a higher frequency of tingling and erythema, effects classically associated with current passage (52, 53). In contrast, nonspecific symptoms, such as concentration difficulties, were slightly more frequent in the sham group, a pattern that may reflect expectation- and attribution-related mechanisms described in the context of nocebo responses (49, 50). This asymmetry supports the interpretation that sensory experiences contribute to treatment credibility, whereas nonspecific symptoms may be amplified in the absence of perceived therapeutic benefit. An additional and clinically informative finding concerns the relationship between adverse event severity and adherence. Greater cumulative adverse event severity was associated with higher protocol engagement, whereas treatment allocation alone did not predict adherence. Although counterintuitive, this association suggests that mild and expected sensory experiences may function as signals of treatment credibility, reinforcing expectancy and sustained engagement rather than acting as barriers. Similar mechanisms have been described in neuromodulation and placebo/nocebo research, in which sensory feedback enhances perceived treatment authenticity and supports continued treatment use (50, 54).

Together, these findings indicate that sensory experiences and patient perceptions play a central role in shaping engagement with autonomous HB-tDCS beyond stimulation assignment alone. Exploratory mediation analyses further indicated that cumulative adverse event severity did not formally mediate the relationship between treatment allocation and adherence. This finding suggests that adverse event burden and adherence operate through partially independent behavioral pathways, reinforcing the interpretation that engagement with autonomous HB-tDCS is shaped predominantly by patient-level experiential and psychosocial factors rather than by stimulation allocation itself.. Importantly, pain catastrophizing was included in mediation models because fibromyalgia severity (FIQ), rather than multidimensional pain index, was used as an adjustment variable, thereby avoiding collinearity between overlapping pain-related constructs**.** This interpretation is consistent with implementation science frameworks emphasizing that engagement with health interventions is driven by experiential, cognitive, and contextual determinants rather than treatment assignment alone (44, 45). When contextualized within the broader fibromyalgia literature, the adherence observed in this study contrasts sharply with adherence to pharmacological treatments, which frequently falls below 35% due to polypharmacy, adverse effects, and limited long-term efficacy (55, 56). Achieving adherence rates exceeding 80% in a population characterized by long-standing symptoms and high psychosocial burden underscores the practical relevance of HB-tDCS as a scalable non-pharmacological intervention.

Consistent with these findings, a recent systematic review and meta-analysis of home-based tDCS for chronic pain reported high adherence and acceptability across heterogeneous protocols and clinical populations, despite substantial variability in stimulation parameters and delivery models (47). This convergence suggests that engagement with HB-tDCS is not primarily driven by technical characteristics of stimulation. Importantly, however, this meta-analysis did not examine patient-level clinical or psychosocial determinants of adherence, nor did it explore experiential factors, symptom burden, or adverse event perception shape engagement. By explicitly modeling these dimensions, the present study addresses a critical gap in the existing literature and extends prior evidence by identifying behavioral and psychosocial factors associated with adherence under fully autonomous HB-tDCS conditions. Comparisons with previous HB-tDCS studies further emphasize the relevance of these findings. While remotely supervised or technician-mediated protocols often report high feasibility, they rely on continuous technical support and real-time supervision. In contrast, the present study evaluated adherence under fully autonomous conditions, imposing greater demands on patient self-management and thereby offering higher ecological validity. The high adherence observed under these conditions is therefore particularly meaningful for real-world scalability and routine clinical implementation (51, 57). From an implementation perspective, these findings support fully autonomous HB-tDCS as a viable adjunctive strategy for chronic pain services, particularly in settings with limited access to specialized care, where scalability, safety, and sustained patient engagement are essential. Features such as pre-programmed sessions, impedance monitoring, and standardized electrode positioning facilitate safe self-administration while preserving professional prescription and control, thereby balancing patient autonomy with clinical oversight.

Several limitations should be acknowledged. *First*, the exclusive inclusion of women limits the generalizability of these findings to male patients with fibromyalgia, who may differ in psychosocial profiles, and treatment engagement. Although fibromyalgia has a markedly higher prevalence in women, future studies should specifically examine adherence and safety of autonomous HB-tDCS in more gender-diverse samples. *Second*, although adherence was objectively verified using device-generated logs, adverse events were self-reported, which may introduce bias related to mood, expectancy, or symptom vigilance. However, this limitation is mitigated by using standardized CTCAE classifications and objective adherence metrics, allowing a clear distinction between subjective reporting and behavioral engagement. *Third*, despite rigorous training and monitoring procedures, adherence behavior in routine clinical practice may differ from that observed under the structured conditions of a clinical trial. *Fourth*, although impedance monitoring and programmable lock systems enhanced safety and protocol fidelity, these technical features may add complexity and pose challenges for large-scale implementation in routine care. Finally, while the protocol was designed to approximate real-world practice, longer-term studies are needed to assess adherence and safety in maintenance or extension protocols and in more heterogeneous populations. Importantly, a key strength of the present study is that the device was specifically developed for home-based use, enabling patient self-administration without the need for continuous remote supervision. This approach prioritizes autonomy and scalability, while the programmable system preserves professional prescription and control. This combination of patient independence and clinician oversight represents a critical advantage, safeguarding fidelity and safety while reinforcing the translational relevance of the HB-tDCS model for large-scale implementation within healthcare systems. Finally, longer-term studies are required to evaluate adherence and safety beyond the treatment period and in more heterogeneous clinical populations.

In conclusion, fully autonomous HB-tDCS demonstrated high adherence, a favorable safety profile, and strong feasibility in fibromyalgia. Adherence and safety were shaped by distinct clinical and psychosocial factors, underscoring the importance of patient profiling and implementation-oriented design. Together, these findings strengthen the translational potential of HB-tDCS as a scalable non-pharmacological intervention for chronic pain.

## Data Availability

The raw data supporting the conclusions of this article will be made available by the authors, without undue reservation.
