# Supplementary material for: Determinants of adherence and safety in fully autonomous home-based transcranial direct current stimulation for fibromyalgia
Source: Front Pain Res (Lausanne). 2026 Mar 16;7:1773425. doi: 10.3389/fpain.2026.1773425 (PMC13033732; doi:10.3389/fpain.2026.1773425)
Supplement: Supplementary file 1 [file Supplementaryfile1.docx]

**SUPPLEMENTARY MATERIALS (S1)**

**Home-Based tDCS Device**

The first home session was performed under remote supervision, and weekly contacts via WhatsApp were made to monitor progress and ensure protocol adherence. Records of session timing, duration, current intensity, and contact impedance were downloaded for compliance verification, and verified by a biomedical engineer.

The Laboratory of Pain and Neuromodulation developed and validated this home-based tDCS device, ensuring secure usage while monitoring and recording session quality and duration, in collaboration with the Biomedical Engineering department at HCPA in Porto Alegre, Brazil [^8,28^](https://www.zotero.org/google-docs/?tEc5KF). The device is registered with the ANVISA under number Nº80079190028 and has been specifically designed for home use, as demonstrated by its application in various trials conducted by our research group [^13,27,28^](https://www.zotero.org/google-docs/?L6aPsF). This device was developed and validated for use at home, as demonstrated by its previous use in different trials conducted by our group [^13,27,28^](https://www.zotero.org/google-docs/?VG9qHL). The device monitors contact impedance at a sampling rate of 1 mA and interrupts the session if the impedance exceeds a predetermined value of 1 mA for an interval of 5 seconds or if current variation is greater than 10%. The equipment records the time and duration of use, as well as the session time, which allows for adherence monitoring. Since July 2020, the equipment has been commercialized by Mendes and Barbosa Produtos Médicos Ltda Quark Medical (Brazil). The tDCS equipment for home use is presented in Figure 1.


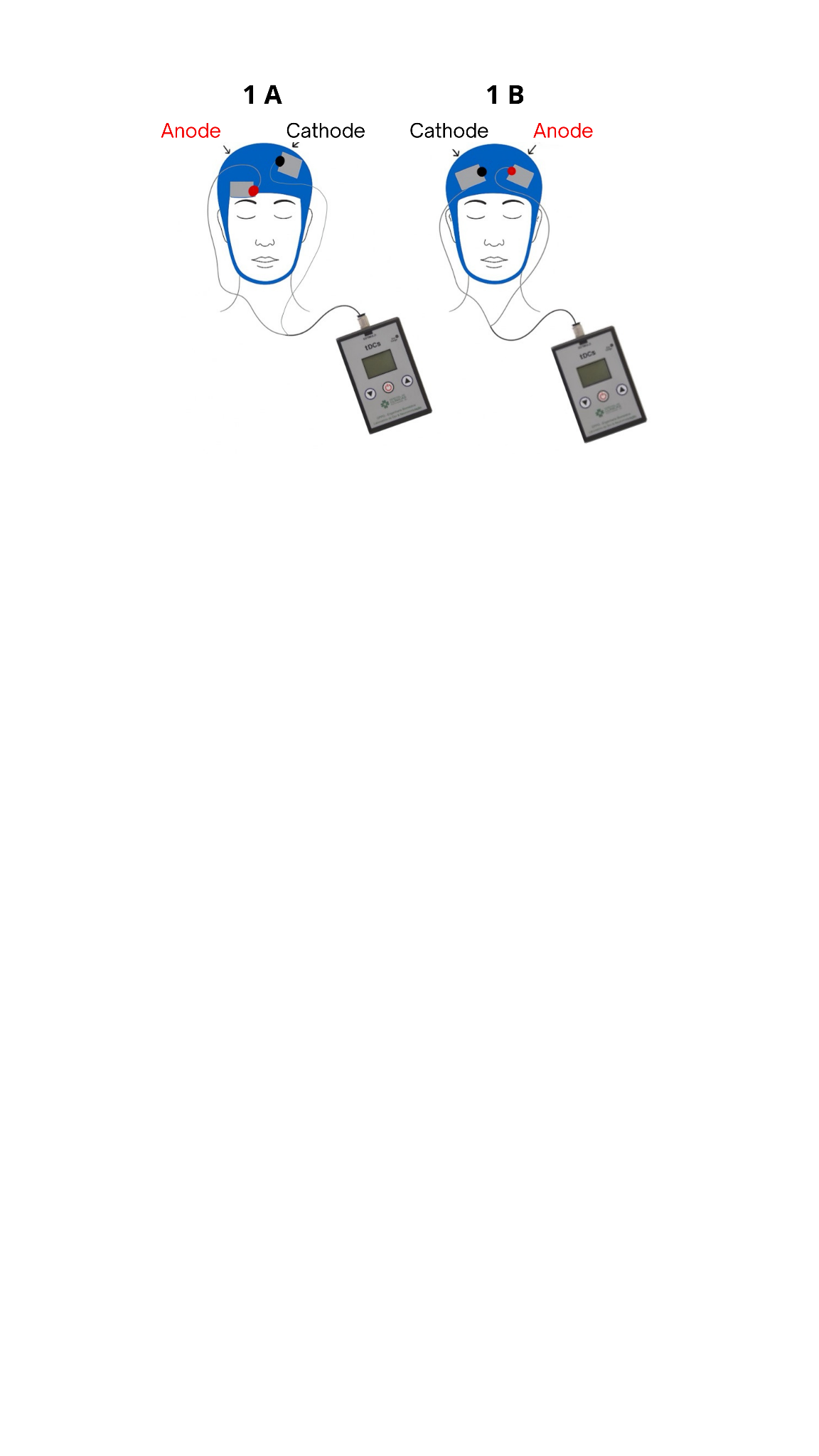


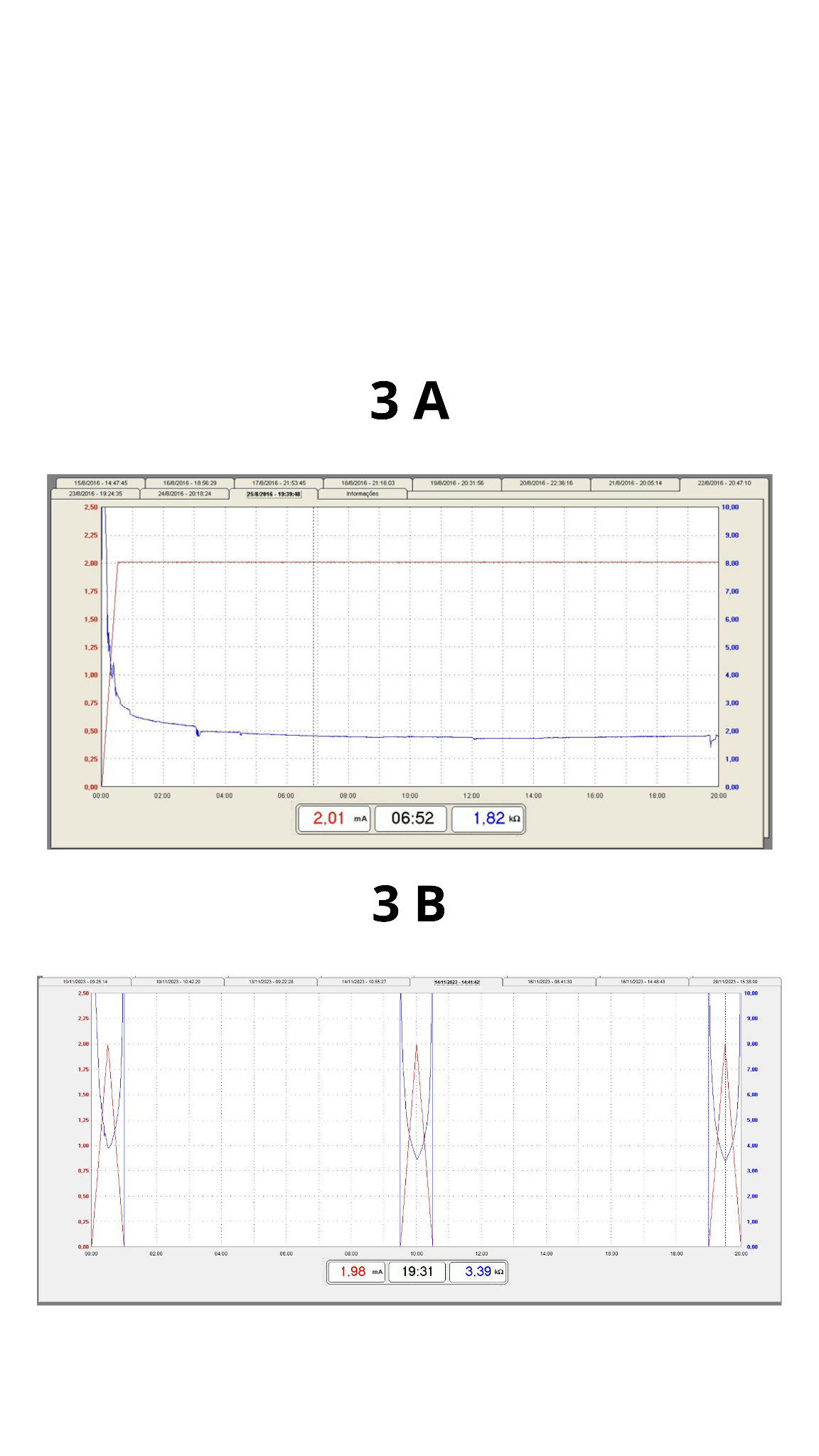

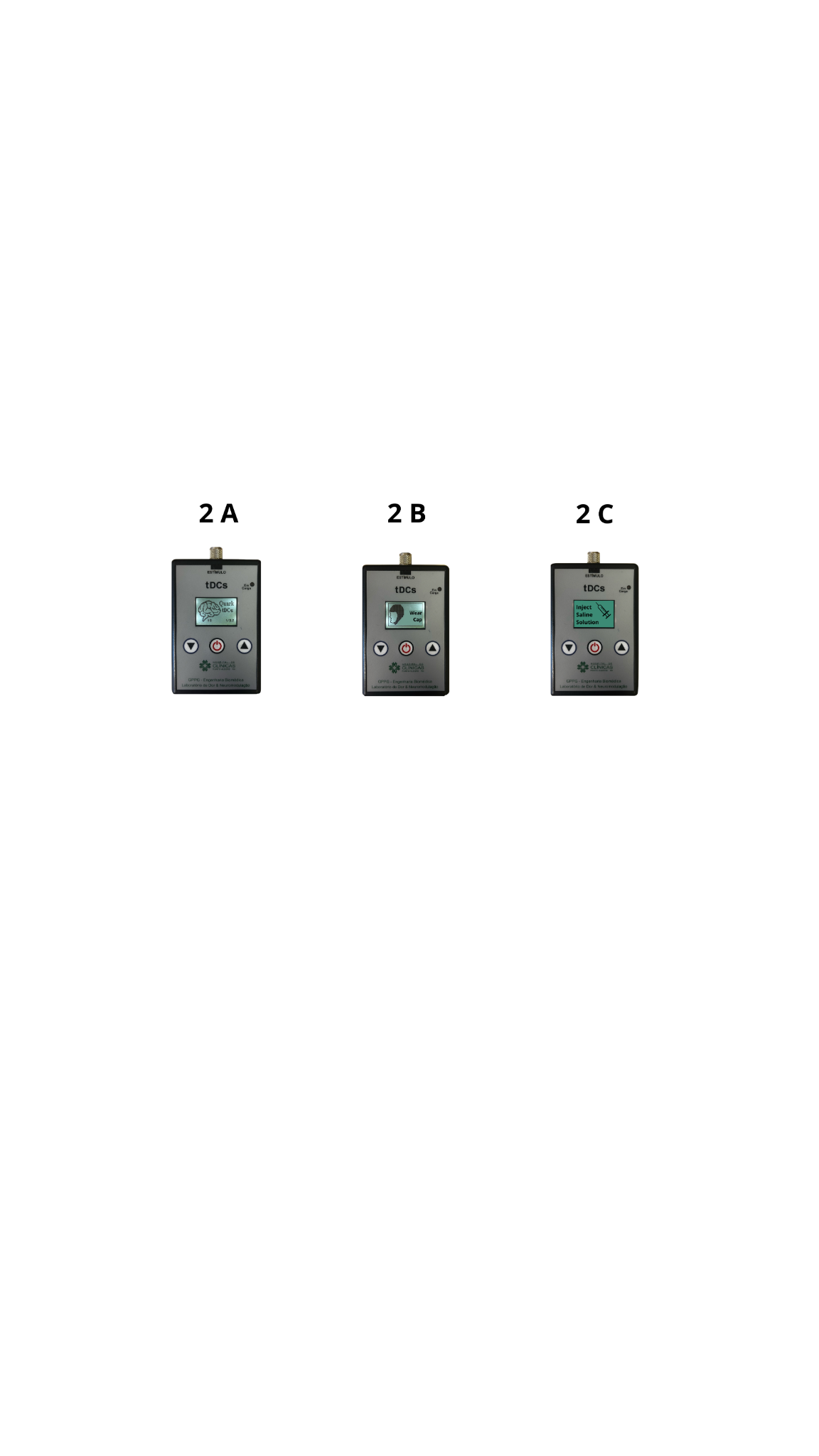


**Figure 1.** Schematic representation of the cap, electrode montage, and device features.

Electrode montages:

1. (A1) anodal tDCS over the left dorsolateral prefrontal cortex (F3) with the cathode over the right DLPFC (F4);
2. (B1) anodal tDCS over M1 (C3) with the cathode over the contralateral supraorbital region (Fp2).

(2) Device features: (2A) display; (2B) cap orientation; (2C) saline solution injection.

(3) Representative curves recorded during stimulation: (3A) current intensity during an active tDCS session; (3B) contact impedance during ramp-up and ramp-down phases (at baseline, 10 min, and 20 min).

**Electrodes position and HB-tDCS stimulation protocol**

This study protocol used scalp electrodes positioned according to the 10-20 system for EEG: anode at F3 and cathode at F4, or anode at C3 and cathode at Fp2 (see Figure 1A, 1B). Active tDCS was applied at 2 mA for 20 min, with 20 s ramp-up/ramp-down and sham sessions mimicked stimulation with 30 s of current at the beginning, 10, and 20 min (see Figure 3). This protocol was designed to mimic active tDCS but without delivering sustained stimulation. To maintain blinding, the same montage and automatic device programming was used for both a-tDCS and s-tDCS. The equipment was programmed to provide a predetermined number of sessions and to prevent the occurrence of a new session before 16 h. This protocol was designed to ensure that participants did not receive more tDCS sessions than the programmed, potentially leading to adverse effects or interfering with the study. The electrodes are 35cm2 and coated with a vegetable sponge moistened with saline solution (administered by two silicone cannulas coupled to the electrode), they were used with a neoprene elastic cap available in varied sizes for proper adjustment to the patients' heads. To ensure the blinding of participants regarding the intervention they received the device was programmed to automatically turn on and off at each of the specified time points for both active and s-tDCS conditions. This approach was adopted to ensure that participants were unaware of whether they were receiving active or sham stimulation at any given time during the study. The device presented a warning in case of poor contact, instructing to adjust the cap or inject extra saline solution (Figure 1 - 2C).

The study’s protocol included visits to the center for baseline assessment, training on how to use the device, and assessment at treatment end. The study was conducted at the Center for Clinical Research at Hospital de Clínicas de Porto Alegre, affiliated with the Federal University of Rio Grande do Sul (UFRGS), Porto Alegre, Brazil. The treatment protocol for tDCS at home involved several steps to ensure proper use and adherence to the protocol.

**
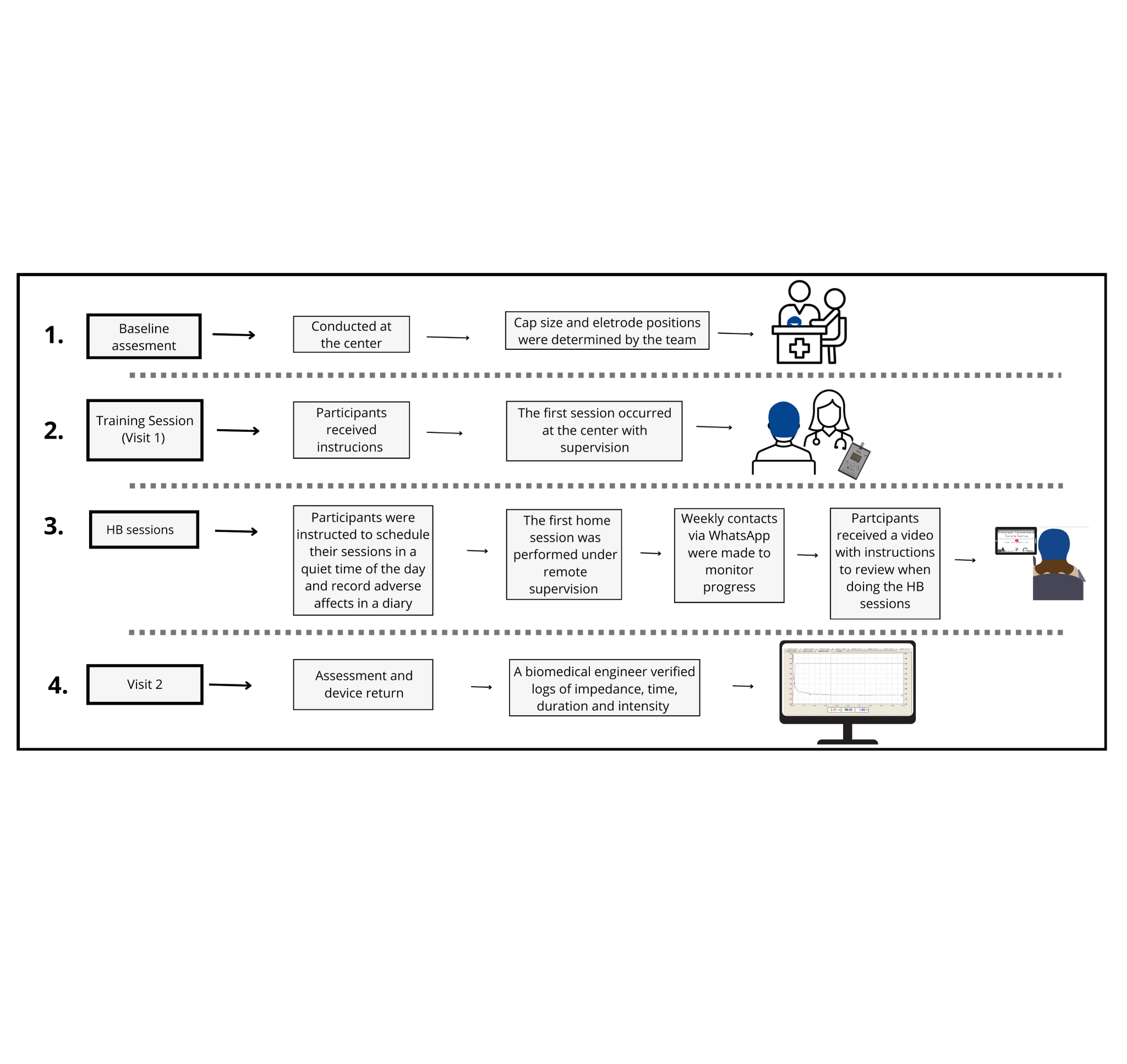
**

**Figure 2.** Structured sequence of the study's protocol: (1) Baseline assessment with instructions at the center; (2) Visit 1 when the first session occurred; (3) Home-based session with contact via WhatsApp and support via video; (4) Visit 2 with return of device and assessments

**The RCTs' protocols consisted of a structured sequence:**

**Baseline Assessment**: This began at the center, where participants were initially assessed.

**Training Session (Visit 1):** Volunteers received comprehensive training on using the tDCS device correctly. They were provided with instructions for self-administration at home. The cap size and electrode positions were determined, and participants were given a link to a video guide for self-administration *(https://youtu.be/3Wtji4esOGE).* They were also encouraged to contact the research team if needed.

**Visit 2:** Occurred four weeks after starting the tDCS protocol. Participants returned to the center for a treatment-end assessment and returned the device. Throughout the protocol, participants were required to use the device appropriately and keep records of any adverse effects experienced.

Electrode Positioning: Cap size was determined by head circumference. Electrodes were inserted into sponges following randomization, with anode marked red and cathode black to avoid placement errors.

Training and Self-Administration: The first session was supervised. Participants were instructed to (i) clean the skin with alcohol, (ii) position the cap seam at the midline, (iii) fill syringes with ~6 ml of saline, and (iv) check for skin irritation.

Compliance Monitoring: Participants recorded adverse effects in a diary. A biomedical engineer verified logs of impedance, time, duration, and intensity. Acceptable impedance was 8–4 kΩ at onset and 3–2 kΩ during stimulation, with ≥10 min at this target range considered valid. This is supported by studies showing 10-min tDCS effectiveness in inducing neuroplasticity (Nitsche, 2000; Bikson, 2016) [2,14].

***Cap Size and Electrode Positioning***

The process of determining cap size and electrode positions involved several steps:

1. Measuring the participant's head circumference to select the appropriate cap size (small, medium, or large).
2. Participants wore the cap while the researcher identified electrode positions following the 10-20 EEG system.
3. Electrode positioning on the cap was based on randomization, with scenarios such as anode at F3 and cathode at F4 (see Figure 1).
4. Electrodes were inserted into vegetable sponges and secured in the cap following the 10-20 EEG system.
5. To avoid electrode placement errors, the anode was marked red, and the cathode was marked black.
6. Detailed instructions for self-administration of the tDCS device at home can be found here: [*https://www.jove.com/video/57614/home-based-transcranial-direct-current-stimulation-device-development*](https://www.jove.com/video/57614/home-based-transcranial-direct-current-stimulation-device-development)*.*

***Training and Self-Application of tDCS at Home***

The training and instructions for participants to self-administer tDCS at home included the following steps:

1. Participants received initial training on equipment usage and skin irritation identification.
2. The first treatment session was supervised with detailed instructions provided through a step-by-step video guide.
3. Participants were instructed to prepare the stimulation area by exposing it in front of a mirror.
4. The skin under the electrodes was cleaned with alcohol to remove creams, dirt, or grease.
5. Participants were guided to wear the cap, positioning the seam between their eyebrows.
6. Approximately 6 ml of saline was added to the syringes connected to the sponges.

***HB-tDCS Sessions and Compliance Monitoring***

To ensure compliance with protocol during HB-tDCS sessions, we implemented several procedures:

1. Participants were advised to choose a quiet and suitable time in their daily schedule for their treatment session.
2. The initial home-based session was conducted under remote supervision, allowing participants to seek assistance from the research team if needed.
3. A weekly contact through WhatsApp to track participants' progress and ensure they adhered to the treatment protocol.
4. Participants were encouraged to contact the research team for assistance.
5. Participants were instructed to promptly document any adverse effects in a diary following each tDCS home session.

To verify the adherence to the following protocol, a biomedical engineer reviewed the records to confirm the session time, duration, current intensity, and contact impedance. The average impedance was employed to assess the quality of the delivered current. The impedance should range between 8 and 4 kΩ at the beginning of stimulation, with the target during treatment between 3 and 2 kΩ. For a valid 20-minute a-DCS session, impedance levels should be between 3 and 2 kΩ for at least half the time in the case of a duration of 10 min. The literature supports the choice of a 10-minute duration by demonstrating its effectiveness in promoting neuroplasticity, with studies by Nitsche (2000) [^20^](https://www.zotero.org/google-docs/?tKd3sl) and Bikson (2016) [^33^](https://www.zotero.org/google-docs/?hbGkE3) adding to this understanding.

**REFERENCES**

[1. Jones GT, Atzeni F, Beasley M, Flüß E, Sarzi-Puttini P, Macfarlane GJ. The prevalence of fibromyalgia in the general population: a comparison of the American College of Rheumatology 1990, 2010, and modified 2010 classification criteria. *Arthritis Rheumatol Hoboken NJ*. 2015;67(2):568-575. doi:10.1002/art.38905](https://www.zotero.org/google-docs/?kzIMZG)

[2. Desai R, Jo A, Marlow NM. Risk for Medication Nonadherence Among Medicaid Enrollees With Fibromyalgia: Development of a Validated Risk Prediction Tool. *Pain Pract Off J World Inst Pain*. 2019;19(3):295-302. doi:10.1111/papr.12743](https://www.zotero.org/google-docs/?kzIMZG)

[3. Cui Z, Zhao Y, Novick D, Faries D. Predictors of duloxetine adherence and persistence in patients with fibromyalgia. *J Pain Res*. 2012;5:193-201. doi:10.2147/JPR.S31800](https://www.zotero.org/google-docs/?kzIMZG)

[4. Moore RA, Fisher E, Häuser W, et al. Pharmacological therapies for fibromyalgia (fibromyalgia syndrome) in adults ‐ an overview of Cochrane Reviews - Moore, RA - 2021 | Cochrane Library. Accessed September 30, 2024. https://www.cochranelibrary.com/cdsr/doi/10.1002/14651858.CD013151.pub2/full](https://www.zotero.org/google-docs/?kzIMZG)

[5. Macfarlane GJ, Kronisch C, Dean LE, et al. EULAR revised recommendations for the management of fibromyalgia. *Ann Rheum Dis*. 2017;76(2):318-328. doi:10.1136/annrheumdis-2016-209724](https://www.zotero.org/google-docs/?kzIMZG)

[6. Petzke F, Brückle W, Eidmann U, et al. [General treatment principles, coordination of care and patient education in fibromyalgia syndrome : Updated guidelines 2017 and overview of systematic review articles]. *Schmerz Berl Ger*. 2017;31(3):246-254. doi:10.1007/s00482-017-0201-6](https://www.zotero.org/google-docs/?kzIMZG)

[7. Perrot S, Russell IJ. More ubiquitous effects from non-pharmacologic than from pharmacologic treatments for fibromyalgia syndrome: a meta-analysis examining six core symptoms. *Eur J Pain Lond Engl*. 2014;18(8):1067-1080. doi:10.1002/ejp.564](https://www.zotero.org/google-docs/?kzIMZG)

[8. Carvalho F, Brietzke AP, Gasparin A, et al. Home-Based Transcranial Direct Current Stimulation Device Development: An Updated Protocol Used at Home in Healthy Subjects and Fibromyalgia Patients. *J Vis Exp JoVE*. 2018;(137):57614. doi:10.3791/57614](https://www.zotero.org/google-docs/?kzIMZG)

[9. Lefaucheur JP, Wendling F. Mechanisms of action of tDCS: A brief and practical overview. *Neurophysiol Clin Clin Neurophysiol*. 2019;49(4):269-275. doi:10.1016/j.neucli.2019.07.013](https://www.zotero.org/google-docs/?kzIMZG)

[10. Huang Y, Thomas C, Datta A, Parra LC. Optimized tDCS for Targeting Multiple Brain Regions: An Integrated Implementation. *Annu Int Conf IEEE Eng Med Biol Soc IEEE Eng Med Biol Soc Annu Int Conf*. 2018;2018:3545-3548. doi:10.1109/EMBC.2018.8513034](https://www.zotero.org/google-docs/?kzIMZG)

[11. Fregni F, El-Hagrassy MM, Pacheco-Barrios K, et al. Evidence-Based Guidelines and Secondary Meta-Analysis for the Use of Transcranial Direct Current Stimulation in Neurological and Psychiatric Disorders. *Int J Neuropsychopharmacol*. 2021;24(4):256-313. doi:10.1093/ijnp/pyaa051](https://www.zotero.org/google-docs/?kzIMZG)

[12. Polanía R, Nitsche MA, Korman C, Batsikadze G, Paulus W. The importance of timing in segregated theta phase-coupling for cognitive performance. *Curr Biol CB*. 2012;22(14):1314-1318. doi:10.1016/j.cub.2012.05.021](https://www.zotero.org/google-docs/?kzIMZG)

[13. Caumo W, Alves RL, Vicuña P, et al. Impact of Bifrontal Home-Based Transcranial Direct Current Stimulation in Pain Catastrophizing and Disability due to Pain in Fibromyalgia: A Randomized, Double-Blind Sham-Controlled Study. *J Pain*. 2022;23(4):641-656. doi:10.1016/j.jpain.2021.11.002](https://www.zotero.org/google-docs/?kzIMZG)

[14. Charvet LE, Shaw MT, Bikson M, Woods AJ, Knotkova H. Supervised transcranial direct current stimulation (tDCS) at home: A guide for clinical research and practice. *Brain Stimulat*. 2020;13(3):686-693. doi:10.1016/j.brs.2020.02.011](https://www.zotero.org/google-docs/?kzIMZG)

[15. Brietzke AP, Zortea M, Carvalho F, et al. Large Treatment Effect With Extended Home-Based Transcranial Direct Current Stimulation Over Dorsolateral Prefrontal Cortex in Fibromyalgia: A Proof of Concept Sham-Randomized Clinical Study. *J Pain*. 2020;21(1-2):212-224. doi:10.1016/j.jpain.2019.06.013](https://www.zotero.org/google-docs/?kzIMZG)

[16. Caumo W, Ramos RL, Serrano PV, et al. Efficacy of Home-Based Transcranial Direct Current Stimulation Over the Primary Motor Cortex and Dorsolateral Prefrontal Cortex in the Disability Due to Pain in Fibromyalgia: A Factorial Sham-Randomized Clinical Study. *J Pain*. 2024;25(2):376-392. doi:10.1016/j.jpain.2023.09.001](https://www.zotero.org/google-docs/?kzIMZG)

[17. Valero-Cabré A, Amengual JL, Stengel C, Pascual-Leone A, Coubard OA. Transcranial magnetic stimulation in basic and clinical neuroscience: A comprehensive review of fundamental principles and novel insights. *Neurosci Biobehav Rev*. 2017;83:381-404. doi:10.1016/j.neubiorev.2017.10.006](https://www.zotero.org/google-docs/?kzIMZG)

[18. Moriarty TA, Mermier C, Kravitz L, Gibson A, Beltz N, Zuhl M. Acute Aerobic Exercise Based Cognitive and Motor Priming: Practical Applications and Mechanisms. *Front Psychol*. 2019;10. doi:10.3389/fpsyg.2019.02790](https://www.zotero.org/google-docs/?kzIMZG)

[19. Cardenas-Rojas A, Pacheco-Barrios K, Giannoni-Luza S, Rivera-Torrejon O, Fregni F. Noninvasive brain stimulation combined with exercise in chronic pain: a systematic review and meta-analysis. *Expert Rev Neurother*. 2020;20(4):401-412. doi:10.1080/14737175.2020.1738927](https://www.zotero.org/google-docs/?kzIMZG)

[20. Nitsche MA, Paulus W. Excitability changes induced in the human motor cortex by weak transcranial direct current stimulation. *J Physiol*. 2000;527(Pt 3):633-639. doi:10.1111/j.1469-7793.2000.t01-1-00633.x](https://www.zotero.org/google-docs/?kzIMZG)

[21. Im JJ, Jeong H, Bikson M, et al. Effects of 6-month at-home transcranial direct current stimulation on cognition and cerebral glucose metabolism in Alzheimer’s disease. *Brain Stimulat*. 2019;12(5):1222-1228. doi:10.1016/j.brs.2019.06.003](https://www.zotero.org/google-docs/?kzIMZG)

[22. Boggio PS, Nunes A, Rigonatti SP, Nitsche MA, Pascual-Leone A, Fregni F. Repeated sessions of noninvasive brain DC stimulation is associated with motor function improvement in stroke patients. *Restor Neurol Neurosci*. 2007;25(2):123-129.](https://www.zotero.org/google-docs/?kzIMZG)

[23. Song S, Zilverstand A, Gui W, Li HJ, Zhou X. Effects of single-session versus multi-session non-invasive brain stimulation on craving and consumption in individuals with drug addiction, eating disorders or obesity: A meta-analysis. *Brain Stimulat*. 2019;12(3):606-618. doi:10.1016/j.brs.2018.12.975](https://www.zotero.org/google-docs/?kzIMZG)

[24. Sandran N, Hillier S, Hordacre B. Strategies to implement and monitor in-home transcranial electrical stimulation in neurological and psychiatric patient populations: a systematic review. *J NeuroEngineering Rehabil*. 2019;16(1):58. doi:10.1186/s12984-019-0529-5](https://www.zotero.org/google-docs/?kzIMZG)

[25. Antal A, Terney D, Kühnl S, Paulus W. Anodal Transcranial Direct Current Stimulation of the Motor Cortex Ameliorates Chronic Pain and Reduces Short Intracortical Inhibition. *J Pain Symptom Manage*. 2010;39(5):890-903. doi:10.1016/j.jpainsymman.2009.09.023](https://www.zotero.org/google-docs/?kzIMZG)

[26. Pilloni G, Shaw M, Feinberg C, et al. Long term at-home treatment with transcranial direct current stimulation (tDCS) improves symptoms of cerebellar ataxia: a case report. *J Neuroengineering Rehabil*. 2019;16(1):41. doi:10.1186/s12984-019-0514-z](https://www.zotero.org/google-docs/?kzIMZG)

[27. Serrano PV, Zortea M, Alves RL, et al. The effect of home-based transcranial direct current stimulation in cognitive performance in fibromyalgia: A randomized, double-blind sham-controlled trial. *Front Hum Neurosci*. 2022;16. doi:10.3389/fnhum.2022.992742](https://www.zotero.org/google-docs/?kzIMZG)

[28. Brietzke AP, Antunes LC, Carvalho F, et al. Potency of descending pain modulatory system is linked with peripheral sensory dysfunction in fibromyalgia: An exploratory study. *Medicine (Baltimore)*. 2019;98(3):e13477. doi:10.1097/MD.0000000000013477](https://www.zotero.org/google-docs/?kzIMZG)

[29. Keller S, Bann CM, Dodd SL, Schein J, Mendoza TR, Cleeland CS. Validity of the brief pain inventory for use in documenting the outcomes of patients with noncancer pain. *Clin J Pain*. 2004;20(5):309-318. doi:10.1097/00002508-200409000-00005](https://www.zotero.org/google-docs/?kzIMZG)

[30. Krebs EE, Bair MJ, Damush TM, Tu W, Wu J, Kroenke K. Comparative responsiveness of pain outcome measures among primary care patients with musculoskeletal pain. *Med Care*. 2010;48(11):1007-1014. doi:10.1097/MLR.0b013e3181eaf835](https://www.zotero.org/google-docs/?kzIMZG)

[31. Dworkin RH, Turk DC, Wyrwich KW, et al. Interpreting the clinical importance of treatment outcomes in chronic pain clinical trials: IMMPACT recommendations. *J Pain*. 2008;9(2):105-121. doi:10.1016/j.jpain.2007.09.005](https://www.zotero.org/google-docs/?kzIMZG)

[32. Borges RB, Mancuso ACB, Camey SA, et al. Power and Sample Size for Health Researchers: uma ferramenta para cálculo de tamanho amostral e poder do teste voltado a pesquisadores da área da saúde. *Clin Biomed Res*. 2020;40(4). Accessed May 4, 2023. https://seer.ufrgs.br/index.php/hcpa/article/view/109542](https://www.zotero.org/google-docs/?kzIMZG)

[33. Bikson M, Grossman P, Thomas C, et al. Safety of Transcranial Direct Current Stimulation: Evidence Based Update 2016. *Brain Stimulat*. 2016;9(5):641-661. doi:10.1016/j.brs.2016.06.004](https://www.zotero.org/google-docs/?kzIMZG)

[34. Rondon-Ramos A, Martinez-Calderon J, Diaz-Cerrillo JL, et al. Pain Neuroscience Education Plus Usual Care Is More Effective Than Usual Care Alone to Improve Self-Efficacy Beliefs in People with Chronic Musculoskeletal Pain: A Non-Randomized Controlled Trial. *J Clin Med*. 2020;9(7):2195. doi:10.3390/jcm9072195](https://www.zotero.org/google-docs/?kzIMZG)

[35. Watson JA, Ryan CG, Cooper L, et al. Pain Neuroscience Education for Adults With Chronic Musculoskeletal Pain: A Mixed-Methods Systematic Review and Meta-Analysis. *J Pain*. 2019;20(10):1140.e1-1140.e22. doi:10.1016/j.jpain.2019.02.011](https://www.zotero.org/google-docs/?kzIMZG)

[36. Caneiro JP, Bunzli S, O’Sullivan P. Beliefs about the body and pain: the critical role in musculoskeletal pain management. *Braz J Phys Ther*. 2021;25(1):17-29. doi:10.1016/j.bjpt.2020.06.003](https://www.zotero.org/google-docs/?kzIMZG)

[37. Leventhal H, Phillips LA, Burns E. The Common-Sense Model of Self-Regulation (CSM): a dynamic framework for understanding illness self-management. *J Behav Med*. 2016;39(6):935-946. doi:10.1007/s10865-016-9782-2](https://www.zotero.org/google-docs/?kzIMZG)

[38. Brief Pain Inventory (BPI). MD Anderson Cancer Center. Accessed September 30, 2024. https://www.mdanderson.org/research/departments-labs-institutes/departments-divisions/symptom-research/symptom-assessment-tools/brief-pain-inventory.html](https://www.zotero.org/google-docs/?kzIMZG)

[39. Dworkin RH, Turk DC, Farrar JT, et al. Core outcome measures for chronic pain clinical trials: IMMPACT recommendations. *Pain*. 2005;113(1-2):9-19. doi:10.1016/j.pain.2004.09.012](https://www.zotero.org/google-docs/?kzIMZG)

[40. Turk DC, Dworkin RH, Burke LB, et al. Developing patient-reported outcome measures for pain clinical trials: IMMPACT recommendations. *Pain*. 2006;125(3):208-215. doi:10.1016/j.pain.2006.09.028](https://www.zotero.org/google-docs/?kzIMZG)

[41. FDA C for DE and. The FDA Announces New Prescription Drug Information Format. *FDA*. Published online 2006. Accessed September 30, 2024. https://www.fda.gov/drugs/laws-acts-and-rules/fda-announces-new-prescription-drug-information-format](https://www.zotero.org/google-docs/?kzIMZG)

[42. Marques AP, Santos AMB, Assumpção A, Matsutani LA, Lage LV, Pereira CAB. Validação da versão brasileira do Fibromyalgia Impact Questionnaire (FIQ). *Rev Bras Reumatol*. 2006;46:24-31. doi:10.1590/S0482-50042006000100006](https://www.zotero.org/google-docs/?kzIMZG)

[43. Geisser ME, Palmer RH, Gendreau RM, Wang Y, Clauw DJ. A pooled analysis of two randomized, double-blind, placebo-controlled trials of milnacipran monotherapy in the treatment of fibromyalgia. *Pain Pract Off J World Inst Pain*. 2011;11(2):120-131. doi:10.1111/j.1533-2500.2010.00403.x](https://www.zotero.org/google-docs/?kzIMZG)

[44. Schestatsky P, Stefani LC, Sanches PR, et al. Validation of a Brazilian quantitative sensory testing (QST) device for the diagnosis of small fiber neuropathies. *Arq Neuropsiquiatr*. 2011;69(6):943-948. doi:10.1590/s0004-282x2011000700019](https://www.zotero.org/google-docs/?kzIMZG)

[45. de Oliveira Franco Á, da Silveira Alves CF, Vicuña P, et al. Hyper-connectivity between the left motor cortex and prefrontal cortex is associated with the severity of dysfunction of the descending pain modulatory system in fibromyalgia. *PloS One*. 2022;17(5):e0247629. doi:10.1371/journal.pone.0247629](https://www.zotero.org/google-docs/?kzIMZG)

[46. Soldatelli MD, Siepmann T, Illigens BMW, et al. Mapping of predictors of the disengagement of the descending inhibitory pain modulation system in fibromyalgia: an exploratory study. *Br J Pain*. 2021;15(2):221-233. doi:10.1177/2049463720920760](https://www.zotero.org/google-docs/?kzIMZG)

[47. Granovsky Y. Conditioned pain modulation: a predictor for development and treatment of neuropathic pain. *Curr Pain Headache Rep*. 2013;17(9):361. doi:10.1007/s11916-013-0361-8](https://www.zotero.org/google-docs/?kzIMZG)

[48. Bertolazi AN, Fagondes SC, Hoff LS, et al. Validation of the Brazilian Portuguese version of the Pittsburgh Sleep Quality Index. *Sleep Med*. 2011;12(1):70-75. doi:10.1016/j.sleep.2010.04.020](https://www.zotero.org/google-docs/?kzIMZG)

49. [Caumo W, Antunes LC, Elkfury JL, et al. The Central Sensitization Inventory validated and adapted for a Brazilian population: psychometric properties and its relationship with brain-derived neurotrophic factor. J Pain Res. 2017;10:2109-2122. doi:10.2147/JPR.S131479](https://www.zotero.org/google-docs/?yScMpa)

[50. Gorenstein C, Andrade L. Validation of a Portuguese version of the Beck Depression Inventory and the State-Trait Anxiety Inventory in Brazilian subjects. *Braz J Med Biol Res Rev Bras Pesqui Medicas E Biol*. 1996;29(4):453-457.](https://www.zotero.org/google-docs/?kzIMZG)

[51. Sehn F, Chachamovich E, Vidor LP, et al. Cross-cultural adaptation and validation of the Brazilian Portuguese version of the pain catastrophizing scale. *Pain Med Malden Mass*. 2012;13(11):1425-1435. doi:10.1111/j.1526-4637.2012.01492.x](https://www.zotero.org/google-docs/?kzIMZG)

[52. Jornada MN da, Antunes LC, Alves C, et al. Impact of multiple-session home-based transcranial direct current stimulation (M-HB-tDCS) on eating behavior in fibromyalgia: A factorial randomized clinical trial. *Brain Stimulat*. 2024;17(2):152-162. doi:10.1016/j.brs.2024.02.001](https://www.zotero.org/google-docs/?kzIMZG)

[53. Teixeira PEP, Tavares DRB, Pacheco-Barrios K, et al. Development of a Clinical Prediction Rule for Treatment Success with Transcranial Direct Current Stimulation for Knee Osteoarthritis Pain: A Secondary Analysis of a Double-Blind Randomized Controlled Trial. *Biomedicines*. 2022;11(1):4. doi:10.3390/biomedicines11010004](https://www.zotero.org/google-docs/?kzIMZG)

[54. Kazis LE, Anderson JJ, Meenan RF. Effect sizes for interpreting changes in health status. *Med Care*. 1989;27(3 Suppl):S178-189. doi:10.1097/00005650-198903001-00015](https://www.zotero.org/google-docs/?kzIMZG)
